# Protein synthesis of the pro-inflammatory S100A8/A9 complex in plasmacytoid dendritic cells and cell surface S100A8/A9 on leukocyte subpopulations in systemic lupus erythematosus

**DOI:** 10.1186/ar3314

**Published:** 2011-04-14

**Authors:** Christian Lood, Martin Stenström, Helena Tydén, Birgitta Gullstrand, Eva Källberg, Tomas Leanderson, Lennart Truedsson, Gunnar Sturfelt, Fredrik Ivars, Anders A Bengtsson

**Affiliations:** 1 Department of Laboratory Medicine, Section of Microbiology, Immunology and Glycobiology, Lund University, Sölvegatan 23, 223 62 Lund, Sweden; 2 Department of Clinical Sciences, Section of Rheumatology, Lund University and Skåne University Hospital, Kioskgatan 3, 221 85 Lund, Sweden; 3 Department of Experimental Medical Science, Immunology Group, Lund University, Sölvegatan 19, 221 84 Lund, Sweden

## Abstract

**Introduction:**

Systemic lupus erythematosus (SLE) is an autoimmune disease with chronic or episodic inflammation in many different organ systems, activation of leukocytes and production of pro-inflammatory cytokines. The heterodimer of the cytosolic calcium-binding proteins S100A8 and S100A9 (S100A8/A9) is secreted by activated polymorphonuclear neutrophils (PMNs) and monocytes and serves as a serum marker for several inflammatory diseases. Furthermore, S100A8 and S100A9 have many pro-inflammatory properties such as binding to Toll-like receptor 4 (TLR4). In this study we investigated if aberrant cell surface S100A8/A9 could be seen in SLE and if plasmacytoid dendritic cells (pDCs) could synthesize S100A8/A9.

**Methods:**

Flow cytometry, confocal microscopy and real-time PCR of flow cytometry-sorted cells were used to measure cell surface S100A8/A9, intracellular S100A8/A9 and mRNA levels of S100A8 and S100A9, respectively.

**Results:**

Cell surface S100A8/A9 was detected on all leukocyte subpopulations investigated except for T cells. By confocal microscopy, real-time PCR and stimulation assays, we could demonstrate that pDCs, monocytes and PMNs could synthesize S100A8/A9. Furthermore, pDC cell surface S100A8/A9 was higher in patients with active disease as compared to patients with inactive disease. Upon immune complex stimulation, pDCs up-regulated the cell surface S100A8/A9. SLE patients had also increased serum levels of S100A8/A9.

**Conclusions:**

Patients with SLE had increased cell surface S100A8/A9, which could be important in amplification and persistence of inflammation. Importantly, pDCs were able to synthesize S100A8/A9 proteins and up-regulate the cell surface expression upon immune complex-stimulation. Thus, S100A8/A9 may be a potent target for treatment of inflammatory diseases such as SLE.

## Introduction

Systemic lupus erythematosus (SLE) is an autoimmune disease characterized by inflammation in several organ systems, B cell hyperactivity, autoantibodies, complement consumption and an ongoing type I interferon (IFN) production [1,2]. SLE patients usually have more activated peripheral blood mononuclear cells (PBMCs) in circulation than healthy individuals and there are numerous investigations demonstrating abnormalities in different subpopulations which illustrate the complexity of the pathogenesis in this disease. Increased numbers of plasma cells [3,4], HLA-DR^+ ^T cells [5,6] and decreased numbers of circulating dendritic cells [7,8] have been reported. Pro-inflammatory CD16^+ ^monocytes have been described to be increased in rheumatoid arthritis but are so far not investigated in SLE [9].

The IFN-alpha (IFNα) production in SLE is detectable in serum [10], and over-expression of IFNα-regulated genes, termed the type I IFN signature, has also been demonstrated in PBMCs [11-16] as well as in platelets [17]. In mice, type I IFNs induce lymphopenia through redistribution of the lymphocytes [18] and there is an inverse correlation between serum IFNα and leukocyte count in humans [10]. SLE patients have circulating immune complexes (ICs), which often contain RNA or DNA [19,20]. ICs could be endocytosed by the natural IFNα producing cells, the plasmacytoid dendritic cells (pDCs) and induce IFNα production through Toll-like receptor (TLR) 7 or TLR9 stimulation [21,22], which is considered to have a key role in the pathogenesis of SLE [23]. IFNα has many immunomodulatory functions such as inducing monocyte maturation [24], increasing IFNα production from NK cells [25], prolonging the survival of activated T cells [26] and differentiating B cells to plasma cells [27].

S100A8 and S100A9 are members of the calcium-binding S100-protein family and are released at inflammatory sites by phagocytes as a complex (S100A8/A9; also called calprotectin or MRP8/14) [28]. Several pro-inflammatory properties have been described for the S100A8/A9 complex, such as activation of monocytes [29], amplification of cytokine production [30], regulation of migration of myeloid derived suppressor cells [31] and, as demonstrated recently, a ligand for receptor for advanced glycation end products (RAGE) and TLR4 [32]. Patients with SLE have increased serum levels of S100A8/A9 [33,34] and the concentration correlates with disease activity. Here we have investigated the portion and activation status of several leukocyte subpopulations and measured cell surface S100A8/A9 on these cells, corresponding S100A8 and S100A9 mRNA expression as well as serum levels of S100A8/A9 in healthy controls and SLE patients to learn more about the role of these proteins in SLE.

## Materials and methods

### Patients

SLE patients were recruited from an ongoing prospective control program at the Department of Rheumatology, Skåne University Hospital, Lund, Sweden. Blood samples were taken at their regular visits. Healthy subjects, age-matched to the patients, were used as controls. An overview of clinical characteristics is presented in Tables 1 and 2. Disease activity was assessed using SLEDAI-2K [35]. The following SLE treatments were used at the time point of blood sampling: hydroxychloroquine (*n *= 38), azathioprine (*n *= 17), mycophenolatmofetil (*n *= 11), rituximab (within the last 12 months, *n *= 5), methotrexate (*n *= 4), cyclosporine A (*n *= 3), cyclophosphamide (*n *= 2), chloroquine phosphate (*n *= 1) and intravenous immunoglobulins (*n *= 1). All patients fulfilled at least four American College of Rheumatology (ACR) 1982 criteria for SLE [36]. The study was approved by the regional ethics board (LU 378-02). Informed consent was obtained from all participants.

**Table 1:** Clinical characteristics of the SLE patients at the time point of blood sampling

Characteristics	SLE (*n *= 63)	Control (*n *= 33)
**Age, median (range), years**	42 (19 to 81)	45 (24 to 79)
**Female, %**	94	85
**Male, %**	6	15
**Disease duration, median (range), years**	8 (0 to 45)	-
**SLEDAI score, median (range)**	2 (0 to 18)	-
**Seizure**	0	-
**Psychosis**	0	-
**Organic brain syndrome**	0	-
**Visual disturbance**	1	-
**Cranial nerve disorder**	1	-
**Lupus headache**	0	-
**Cerebrovascular accident**	0	-
**Vasculitis**	3	-
**Arthritis**	8	-
**Myositis**	1	-
**Kidney involvement (urinary cast, hematuria, proteinuria or pyuria)**	14	-
**Mucocutaneous activity (rash, alopecia or mucosal ulcers)**	12	-
**Pleurisy**	0	-
**Pericarditis**	0	-
**Low complement (C3 or C4)**	14	-
**Anti-DNA antibodies**	14	-
**Fever**	2	-
**Thrombocytopenia**	1	-
**Leukopenia**	2	-

Items included in SLE disease activity (SLEDAI) are shown.

**Table 2:** Clinical characteristics of the SLE patients (*n *= 63) according to ACR 1982 criteria

ACR criteria	Portion of patients (%)
**Malar rash**	65
**Discoid rash**	25
**Photosensitivity**	67
**Oral ulcers**	29
**Arthritis**	79
**Serositis**	52
**Renal disease**	48
**Neurological disorder**	6
**Hematological manifestation**	54
**-Leukopenia**	41
**-Lymphopenia**	24
**-Thrombocytopenia**	24
**Immunology**	79
**-a-DNA**	59
**ANA**	100

ACR, American College of Rheumatology; ANA, anti-nuclear antibodies.

### Antibodies and reagents

The following antibodies and reagents were used in the flow cytometry analysis of the patients and the healthy volunteers: anti-CD3-Alexa 647, anti-CD4-APC-Cy7, anti-CD19-Pacific Blue, anti-CD14-PE-Cy7 (all from BioLegend, San Diego, CA, USA), anti-CD3-APC-Alexa Fluor 750, anti-CD8-PE-Cy7, anti-HLA-DR-Alexa Fluor 700, anti-CD20-PE, anti-CD38-PE-Cy5, anti-CD27-Alexa Fluor 700 (all from eBioscience, San Diego, CA, USA), propidium iodide, anti-IgD-FITC, anti-CD16-PE-Cy5, mouse IgG1-FITC (all from BD Biosciences Pharmingen, San Diego, CA, USA), anti-BDCA-1-biotin, anti-BDCA-2-PE (both from Miltenyi Biotec Inc., Auburn, CA, USA), anti-S100A8/A9-FITC (27E10, BMA Biomedicals, Rheinstrasse, Switzerland) and streptavidin Qdot-605 (Invitrogen, Carlsbad, CA, USA).

### Flow cytometry

Blood was drawn into cell preparation tubes (BD Biosciences Pharmingen) and PBMCs were isolated on Lymphoprep™ according to manufacturer's instructions (Axis-Shield PoC AS, Oslo, Norway). PBMCs (1 × 10^6 ^cells) were incubated with 10% mouse serum in phosphate buffered saline pH 7.2 (PBS) at a total volume of 50 μl for 20 minutes at 4°C. The cells were washed in PBS (200 g 2 minutes) and incubated with biotinylated antibodies for 20 minutes at 4°C. The cells were washed twice and then incubated for another 20 minutes at 4°C with dye-conjugated antibodies and streptavidin-Qdot 605. Finally, the cells were washed twice and resuspended in 300 μl PBS before analysis in the FACSAria (BD Biosciences Pharmingen).

### Confocal microscopy

T cells, monocytes and pDCs were isolated with negative isolation kits according to manufacturer's instructions (Miltenyi Biotec Inc). Neutrophils were isolated by density gradient centrifugation on Polymorphprep™ according to the manufacturer's protocol (Axis-Shield PoC AS). B cells and mDCs were isolated using a FACSAria cell sorter. B cells were labeled with Pacific Blue-conjugated CD19 antibodies and mDCs were labeled with both Alexa 700-conjugated HLA-DR antibodies and FITC-conjugated BDCA-1 antibodies. Purified cells (8 × 10^4^) were fixed with 4% paraformaldehyde and permeabilized in 0.2% TritonX-100 (Sigma, St. Louis, MO, USA) before incubated with PE-conjugated anti-S100A8/A9 antibodies (27E10, Santa Cruz Biotechnology, Santa Cruz, CA, USA) for 30 minutes. The cells were washed once in PBS and transferred to a glass slide (In Vitro, Braunschweig, Germany) by cytospin for two minutes at a speed of 1,000 g in a Shandon Cytospin 3 (Life Science International LTD, Cheshire, UK). The cells were analyzed in a LSM 510 META microscope (Carl Zeiss, Göttingen, Germany). Fluorescence was detected with pinhole settings corresponding to one airy unit.

### Immune complex stimulation of plasmacytoid dendritic cells

Isolated pDCs (2 × 10^4 ^cells) were cultured in 100 μl Macrophage-SFM (Invitrogen) supplemented with 20 mM HEPES (Invitrogen), 50 μg/ml Gentamicin (Invitrogen), 2 ng/ml GM-CSF (Leukine^®^; Berlex, Montville, NJ, USA) and 500 U/ml IntronA (SP company, Innishannon, Ireland) and incubated for 20 h at 37°C with 5% CO_2 _and 97% humidity with RNA-containing ICs prepared as described previously [37]. Briefly, anti-ribonuclear protein (RNP)-positive sera were pooled and IgG was purified on a protein G column (Protein G Superose HR 10/2, Pharmacia LKB, Uppsala, Sweden). To create ICs, purified IgG at a concentration of 0.25 mg/ml was mixed with necrotic material from Jurkat cell supernatant at a concentration of 5% (v/v). The cells were washed and resuspended in PBS with anti-CD123-FITC (Miltenyi Biotech) and anti-S100A8/A9-PE (Santa Cruz Biotechnology) antibodies for 30 minutes at 4°C before analyzed by flow cytometry. The ICs used in the experiments contained undetectable amounts (< 40 ng/ml) of S100A8/A9 as measured by the in-house ELISA (data not shown). As a negative control, PE-conjugated mouse IgG1 antibodies were used.

### Serum S100A8/A9 detection

For detection of S100A8/A9, microtitre plates (Maxisorp, Nunc, Roskilde, Denmark) were coated with monoclonal antibody MRP8/14 (27E10, BMA Biomedicals) at a concentration of 5 μg/ml, diluted in PBS at a volume of 100 μl/well, and incubated at 4°C over night. The 27E10 antibody detects a specific epitope of S100A8/A9 which is not exposed on the individual subunits. Between every following step, the plate was washed for three times in PBS containing 0.05% Tween 20. After blocking the plate with 1% BSA in PBS for 1 h, serum samples, diluted 1/100 in sample buffer (0.15 M NaCl, 10 mM HEPES (Invitrogen), 1 mM CaCl_2_, 0.02 mM ZnCl_2_, 0.05% Tween 20 and 0.1% BSA), were added at a final volume of 100 μl/well and incubated for 2 h at room temperature under agitation. Biotinylated anti-MRP8/14 (Abcam, Cambridge, UK), diluted 1/2000 in sample buffer, were added at a volume of 100 μl/well, and incubated at 4°C over night. Bound MRP8/14 antibody was detected with alkaline-phosphatase-labelled streptavidin (Dako, Glostrup, Denmark) diluted 1/1000 in sample buffer. After incubation for 1 h at room temperature under agitation, the enzymatic reaction was developed with 1 mg/ml disodium-*p*-nitrophenyl phosphate (Sigma) dissolved in 10% (w/v) dietanolamine pH 9.8 containing 50 mM MgCl_2 _and the absorbance was measured at 405 nm. S100A8/A9 content of one serum sample was quantified using a commercial S100A8/A9 kit (BMA Biomedicals) and used as an internal control. The values reported are means of duplicates with the background subtracted and the concentrations were calculated from titration curves obtained from a pool of normal human serum.

### Real-time PCR

Isolated PBMC from healthy donors were stained with anti-CD3-Alexa 647, anti-CD19-Pacific Blue, anti-CD14-PECy7, anti-CD16-FITC, anti-HLA-DR-Alexa 700, anti-BDCA-1-biotin and anti-BDCA-2-PE antibodies and sorted on a FACSAria before frozen at -80°C in lysis buffer. Total RNA was extracted by Purelink RNA mini Kit (Invitrogen, Carlsbad, CA, USA) and reversely transcribed to cDNA by SuperScript II Platinum synthesis system (Invitrogen) according to manufacturer's instructions. Ribosomal protein L4 (RPL4), S100A8 and S100A9 mRNA were quantified by real-time PCR using the SYBR GreenER kit (Invitrogen) in a MYIQ PCR machine (Bio-Rad, Hercules, CA, USA). The threshold cycle number and levels of each mRNA were determined using the formula 2(Rt-Et), where Rt is the threshold cycle for the housekeeping gene RPL-4 and Et the threshold cycle for the gene of interest.

### Statistics

Data were evaluated with analysis of variance (ANOVA) when comparing healthy controls with SLE patients or within the patient cohort when evaluating different disease manifestations. SAS version 9.2 for Windows XP (SAS Institute Inc., Cary, NC, USA) was used in the statistical evaluation. Correlations were calculated by Spearman rank correlation test without adjustment for multiple testing. All *P*-values were considered significant at *P *< 0.05.

## Results

### SLE patients have more activated leukocytes

First, we wanted to confirm abnormalities in PBMCs from SLE patients described by others to validate our patient material. Over all, SLE patients had markedly decreased cell density of PBMCs as compared to healthy controls (median SLE: 0.41 × 10^6 ^(0.40 to 0.51) cells/ml and median healthy controls 1.00 × 10^6 ^(0.86 to 1.12) cells/ml, *P *< 0.0001). A summary of all investigated leukocyte populations is shown in Table 3. SLE patients had more activated cells with increased HLA-DR expressing CD4^+ ^T cells (*P *= 0.001), CD8^+ ^T cells (*P *= 0.02), pro-inflammatory CD16^+ ^monocytes (*P *= 0.003) and percentage of plasma cells (*P *< 0.0001), as compared to healthy controls. Altogether we have demonstrated that SLE patients have more activated leukocytes as compared to healthy controls and these results are in concordance with previous findings [3,5].

**Table 3:** Frequencies of different cell populations in SLE patients and healthy controls

Cell population	**Healthy controls (median and 95% CI)**^**1**^	SLE patients (median and 95% CI)	*P*-value
**CD3^+ ^T cells**	60.9 (57.8 to 63.9)	65.1 (55.7 to 64.3)	0.42
**CD4^+ ^T cells**	62.1 (57.0 to 64.7)	55.2 (51.3 to 58.9)	0.06
**CD4^+^HLA-DR^+^**	6.5 (5.3 to 7.2)	31.3 (24.9 to 36.9)	0.001
**CD8^+ ^T cells**	15.7 (14.3 to 18.8)	20.4 (18.6 to 24.0)	0.02
**CD8^+^HLA-DR^+^**	40.8 (39.0 to 43.2)	36.9 (33.4 to 41.5)	0.33
**BDCA-1^+ ^mDCs**	0.40 (0.34 to 0.46)	0.31 (0.28 to 0.62)	0.19
**BDCA-2^+ ^pDCs**	0.07 (0.07 to 0.11)	0.09 (0.09 to 0.16)	0.39
**CD14^++^CD16^- ^monocytes**	10.1 (9.6 to 13.3)	16.9 (16.0 to 25.2)	0.013
**CD14^++^CD16^+ ^monocytes**	0.17 (0.15 to 0.27)	0.30 (0.44 to 0.81)	0.003
**CD19^+ ^B cells**	5.6 (4.7 to 6.3)	5.1 (5.0 to 7.8)	0.78
**CD19^+^CD27^+^IgD^-^CD38^+^CD20^-^**	0.43 (0.35 to 0.75)	1.0 (0.8 to 3.0)	< 0.0001

^1^For CD3^+ ^T cells, dendritic cells, monocytes and B cells the percentage is calculated against the total leukocyte count. For all subpopulations, the percentage is calculated against the main population (for example, CD4^+ ^T cells for all CD4^+ ^T cell analyses).

CI, confidence interval; HLA, human leukocyte antigen; mDC, myeloid dendritic cell; pDC, plasmacytoid dendritic cell

### S100A8/A9 is detected on several different cell populations

**Figure 1:**
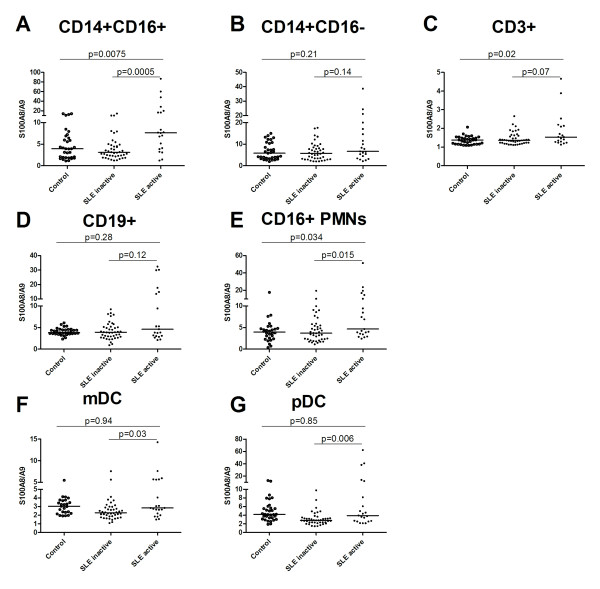
**Increased cell surface S100A8/A9 in patients with active disease compared with inactive disease and healthy controls**. The cell surface S100A8/A9 was determined by flow cytometry on **A**) CD14^++^CD16^+^, **B**) CD14^++^CD16^-^, **C**) CD3^+^, **D**) CD19^+^, **E**) CD16+ PMNs, **F**) BDCA-1^+ ^and **G**) BDCA-2^+ ^cells. The expression is defined as the mean fluorescence index (MFI) ratio between the S100A8/A9 antibody and its control isotype antibody in each experiment. The line represents the median-value. Active disease was defined as SLEDAI > 4 (SLE active).

Detection of S100A8/A9 has previously been demonstrated on the surface of monocytes [32] as well as intracellularly in polymorphonuclear cells [28]. We wanted to see if S100A8/A9 was also present on other cells since our main objective was to investigate possible aberrant expression in SLE. We could detect S100A8/A9 on naïve as well as pro-inflammatory monocytes, PMNs, B cells, myeloid dendritic cells (mDCs) and pDCs in both SLE patients and healthy controls (Figure 1). However, S100A8/A9 could not be detected on T cells where the mean fluorescence index (MFI) ratio indicated little or no cell surface S100A8/A9. Levels of the cell surface S100A8/A9 correlated well in samples from the same individual between the different cell populations (data not shown). Treatment with immunosuppressive drugs at the time of blood sampling was not statistically significantly associated with altered cell surface S100A8/A9. Cell surface S100A8/A9 was not significantly increased in SLE patients as a whole when compared to healthy controls on any cell population investigated (data not shown). We then investigated if cell surface S100A8/A9 was altered in patients with active disease (defined as SLEDAI ≥4) as compared to patients with inactive disease (SLEDAI < 4). Patients with active disease had increased cell surface S100A8/A9 on their CD16^+ ^pro-inflammatory monocytes, pDCs, mDCs as well as PMNs as compared to SLE patients with inactive disease (*P *= 0.0005, *P *= 0.006, *P *= 0.03 and *P *= 0.015, respectively) and increased cell surface S100A8/A9 on their CD16^+ ^pro-inflammatory monocytes and PMNs as compared to healthy controls (*P *= 0.0065 and *P *= 0.034, respectively, Figure 1). Thus we could demonstrate that cell surface S100A8/A9 was associated with disease activity and increased in patients with active disease.

### S100A8 and S100A9 are not produced by all leukocytes

**Figure 2:**
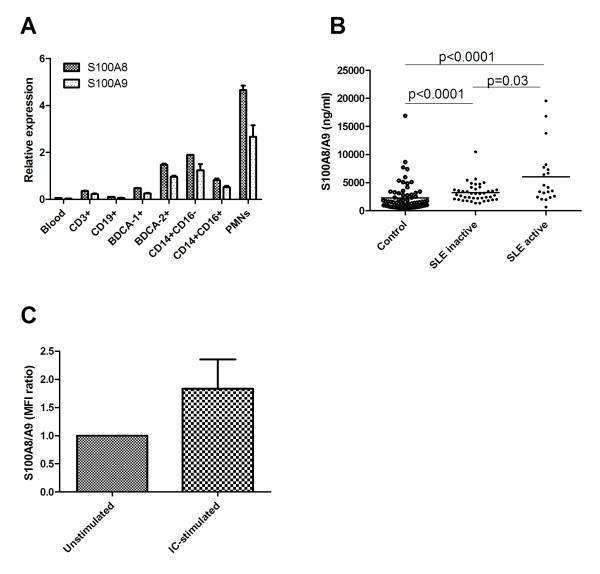
**S100A8/A9 mRNA expression in different cell populations, serum levels and cell surface S100A8/A9 upon pDC activation**. **A**) The relative expression of S100A8 and S100A9 mRNA in different cell populations. PBMCs were isolated and sorted by flow cytometry before determining the mRNA levels of S100A8 and S100A9 by real-time PCR. **B**) Serum levels of S100A8/A9 measured by an in-house ELISA in SLE patients and healthy controls. The line represents the median value. **C**) Purified pDCs were stimulated with immune complexes for 20 h and analyzed for cell surface S100A8/A9 by flow cytometry. The data are presented as the mean fluorescence index (MFI) ratio with one standard deviation as compared to unstimulated cells for each experiment.

**Figure 3:**
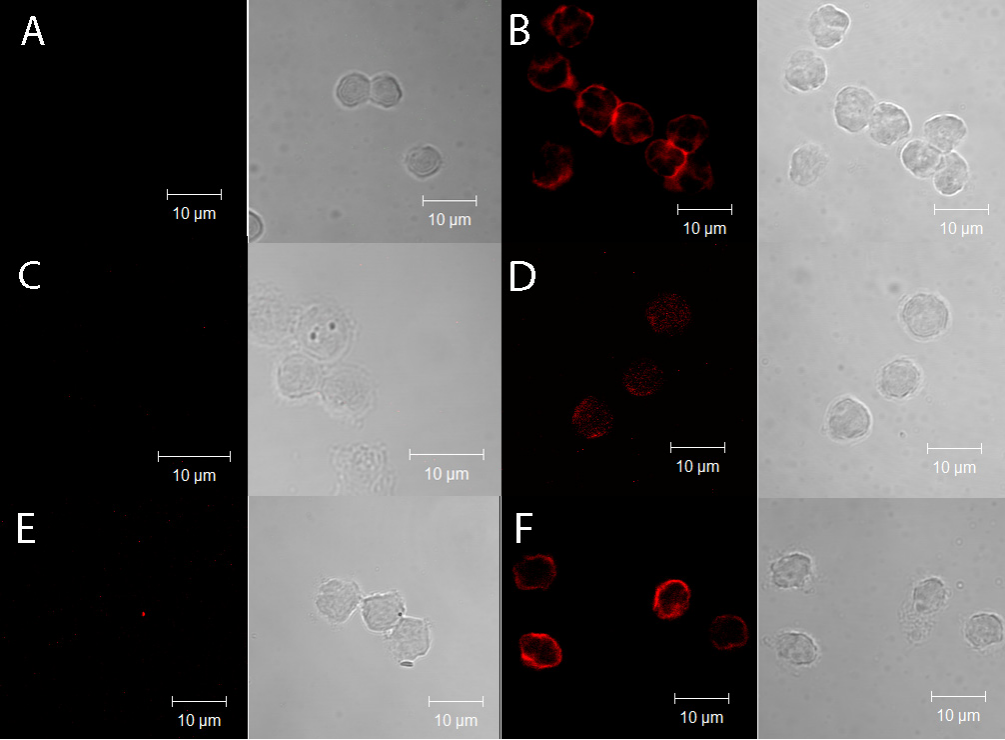
**Analysis of S100A8/A9 staining in different leukocyte populations by confocal microscopy**. Cells were isolated, fixed, permeabilized and stained for S100A8/A9 with the 27E10 antibody. T cells, B cells and mDCs (**A**, **C **and **E**, respectively) had no detectable S100A8/A9 expression while PMNs, pDCs and monocytes (**B**, **D **and **F**, respectively) had membrane-associated, as well as intracellular, S100A8/A9.

Since S100A8/A9 was observed on most cell populations we wanted to know if this was due to expression of the S100A8 and S100A9 genes or due to deposition from external sources. To test the first possibility S100A8 and S100A9 mRNA levels were analyzed in FACS-sorted cells. Only low mRNA levels were found in T cells, B cells and mDCs, despite detection of cell surface S100A8/A9 on B cells and mDCs. However, clearly detectable levels of both S100A8 and S100A9 mRNA were found in monocytes, PMNs and pDCs (Figure 2A). These results confirm that S100A8/A9 is mainly produced by monocytes, PMNs and also by pDCs and the cell surface S100A8/A9 on other cell populations is most likely due to external deposition. We could confirm our flow cytometry data using confocal microscopy where we could detect membrane-associated S100A8/A9 on PMNs, monocytes and pDCs but not on T cells, mDCs or B cells (Figure 3). However, when using a non-confocal setting both mDCs and B cells had a weak S100A8/A9 staining whereas T cells still were negative (data not shown). In addition, we could detect S100A8/A9 with intracellular location in PMNs, monocytes and pDCs further supporting S100A8/A9 protein synthesis by these cells (Figure 3). Altogether, these data demonstrate that besides monocytes and PMNs, pDCs are also able to produce S100A8/A9.

### Increased levels of serum S100A8/A9 in SLE

If the presence of S100A8/A9 on the cell surface were due to external deposition, the level of cell surface S100A8/A9 might correlate with S100A8/A9 serum concentration. We found that S100A8/A9 serum concentrations were clearly increased in SLE as compared to healthy controls (*P *< 0.0001, Figure 2B), in accordance with previous published results [33,34]. Furthermore, we also found the most pronounced increased serum concentrations of S100A8/A9 in patients with arthritis (*P *= 0.016), as was previously reported [33], as well as in patients with kidney involvement (*P *= 0.026). There was also a statistically significant correlation between serum concentration of S100A8/A9 and SLEDAI (*P *< 0.0001, r = 0.49). However, serum S100A8/A9 level correlated only with cell surface S100A8/A9 in PMNs (*P *= 0.027, r = 0.28) and not cell surface S100A8/A9 levels on other leukocyte subpopulations. Thus, we could demonstrate that SLE patients had increased serum levels of S100A8/A9 which correlated to disease activity, but there were no strong correlations between S100A8/A9 cell surface levels and serum levels.

We also investigated whether it was possible to deposit S100A8/A9 on leukocytes *in vitro*. Neither recombinant S100A8/A9 nor serum containing high concentrations of S100A8/A9 (> 5,000 ng/ml) gave any increased surface staining of S100A8/A9 (data not shown). This might suggest that the S100A8/A9 binding ligands were already saturated and that other mechanisms could also be involved in the deposition of S100A8/A9 on the cell surface.

### Increased pDC cell surface S100A8/A9 upon activation

The flow cytometry data in combination with mRNA expression and confocal microscopy data strongly supported that monocytes, PMNs and also pDCs could produce S100A8/A9. Since the pDC is central in SLE pathogenesis and S100A8/A9 production is, to our knowledge, previously only described in monocytes and PMNs, we wanted to further investigate this subpopulation. When stimulating isolated pDCs with ICs in a serum-free medium the cell surface S100A8/A9 increased (Figure 2C) supporting that pDCs are able to synthesize S100A8/A9 and actively transport S100A8/A9 to the cell surface upon activation. Thus, pDCs could up-regulate cell surface S100A8/A9 in response to activation and we propose that pDCs are also able to synthesize S100A8/A9 proteins.

## Discussion

SLE is a heterogeneous disease with involvement of virtually all organ systems, including the skin, joints and kidney. T and B cell activation, production of autoantibodies, formation of ICs and the subsequent tissue damage if the immune complexes are not handled correctly are all well described important events in the SLE pathogenesis. Also, many other cell populations besides T and B cells are activated as demonstrated in this as well as in many other studies [3-8,38]. We found that SLE patients had an increased percentage of plasma cells as well as increased expression of HLA-DR on their T cells as demonstrated previously [4-6]. Pro-inflammatory CD16^+ ^monocytes have increased potential to produce pro-inflammatory cytokines such as TNFα [39] and are increased in inflammatory diseases, such as rheumatoid arthritis [9]. We could demonstrate an increased percentage of CD16^+ ^pro-inflammatory monocytes also in SLE. Altogether, we have seen pathological changes with increased activation of B cells, T cells and monocytes in SLE patients and it should be noted that these very clear cut pathological changes were also seen in many patients with low disease activity. Although similar observations have been reported by others, our cellular analyses serve as a validation of methods and patient material used in this study. Also, it is important to assess numbers and activation status of the different leukocyte populations that we investigate for S100A8/A9 expression.

Despite the lack of S100A8 and S100A9 mRNA in many leukocyte subpopulations, cell surface S100A8/A9 was detected by flow cytometry on all of the investigated cell populations except for T cells. It is known that the S100A8/A9 complex is produced by phagocytes such as monocytes and neutrophils [28] and we could verify by mRNA analyses that, among the cell populations studied, only monocytes, PMNs and also pDCs could produce S100A8 and S100A9. To our knowledge it has not previously been shown that pDCs are able to produce S100A8/A9. Interestingly, S100A8/A9 seemed to be actively transported to the outside of the membrane upon pDC activation suggesting that the cell surface S100A8/A9 on pDCs indeed could have biological functions. The exact function of S100A8/A9 production in this particular cell remains to be shown, but the pDCs as an IFNα producing cell is clearly central in the pathogenesis of SLE. S100A8/A9 has been suggested to have several important functions in the immune system such as activation of monocytes, migration of myeloid derived suppressor cells and amplification of cytokine production [29-31,40]. Furthermore, cells from S100A9 deficient mice display reduced TNFα production when stimulated with LPS, a deficiency that could be restored by addition of extracellular S100A8/A9 [29]. Also, the S100A8/A9 complex can increase TNFα production upon LPS stimulation [29]. Recently, Björk *et al. *[32] described that quinoline-3-carboxamides or antibodies against S100A9 could inhibit the LPS-induced TNFα production. Recently, Loser *et al *demonstrated that S100A8 and S100A9 are crucial for the development of autoreactive CD8^+ ^T cells and systemic autoimmunity in a mouse model [41]. Altogether, this illustrates that S100A8/A9 could serve as an amplifier of inflammation and should thus be regarded as a potential target for treatment of inflammatory diseases such as SLE.

Increased serum levels of S100A8/A9 in SLE, as well as in other connective tissue diseases such as rheumatoid arthritis and Sjogren's syndrome, was first described in 1990 by Kuruto *et al. *[42] and was later confirmed in SLE both in serum and by a proteomic-based study on PBMCs [33,34,43]. We could demonstrate increased serum concentrations of S100A8/A9 in SLE patients as compared to healthy controls and a correlation to disease activity. Cell surface S100A8/A9 was also increased on several leukocyte subpopulations such as pDCs in patients with active disease as compared to patients with inactive disease. However, the cell surface S100A8/A9 on some leukocyte subpopulations such as B cells and mDCs could not be explained by protein synthesis. Furthermore, it has previously been demonstrated that endothelial cells were coated with cell surface S100A8/A9 but lacked mRNA expression of these genes [44]. Clearly, other mechanisms also explain why S100A8/A9 are present on cell surfaces such as ligand up-regulation and deposition of S100A8/A9 from other sources such as serum or neutrophil extracellular traps (NET), which have a high content of S100A8/A9 [45]. High serum levels were, however, not generally associated with high cell surface S100A8/A9 levels. Furthermore, incubation of S100A8/A9 rich serum with leukocytes expressing low levels of S100A8/A9 on their surface could not increase the cell surface S100A8/A9 suggesting that the ligands most likely were already saturated. The broad binding pattern of S100A8/A9 to many cell populations indicates a general binding partner, and heparan sulfate glycosaminoglycans (GAG) structures and carboxylated glycans have been reported to bind to the S100A8/A9 complex as well as to the homodimer S100A9/A9 [44,46]. The low level or absence of cell surface S100A8/A9 on T cells would then suggest a specific GAG-structure epitope not present on T cells but otherwise commonly expressed on most leukocyte populations.

## Conclusions

Here we could demonstrate the presence of S100A8/A9 on monocytes and PMNs as well as pDCs, mDCs and B cells, which are all cells that are important in the inflammatory response in SLE. We could demonstrate that pDCs, a cell population believed to be central in the SLE pathogenesis, could synthesize S100A8/A9 and express this protein on its surface upon activation. However, the exact function of S100A8/A9 is not fully understood and needs further studies. In fact, there are ongoing clinical trials in SLE performed by us using a quinoline-3-carboxamide compound targeting S100A9 which will give us more information on the role of S100A9 blockade in SLE and if it can be used as a therapeutic target.

## Abbreviations

GAG: glycosaminoglycans; IC: immune complex; IFN: interferon; mDC: myeloid dendritic cell; MFI: mean fluorescence index; NET: neutrophils extracellular trap; PBMC: peripheral blood mononuclear cell; pDC: plasmacytoid dendritic cell; PMN: polymorphonuclear neutrophil; RAGE: receptor for advanced glycation end products; RNP: ribonuclear protein; SLE: systemic lupus erythematosus; TLR: toll like receptor.

## Competing interests

FI holds research grants from Active Biotech AB. AB holds research grants and consulting fees from Active Biotech AB. MS is an employee of Active Biotech AB. TL is a part-time employee of Active Biotech AB. TL holds shares and stock options in Active Biotech AB. Active Biotech AB develops S100A9-binding compounds for the treatment of autoimmune diseases.

## Authors' contributions

CL carried out some of the flow cytometry, the confocal microscopy, pDC isolation and stimulation, performed the statistical analyses, participated in the design of the study and drafted the manuscript. MS carried out some of the flow cytometry and participated in the design of the study. HT and BG performed some of the ELISA analyses and revised the manuscript. EK performed the real-time PCR. TL, LT and GS participated in the design of the study and critically revised the manuscript. AB participated in the design of the study and helped to draft the manuscript and supervised the project. All authors participated in the design of the study, revised and approved the final manuscript.
